# The outcomes of pregnant and postpartum patients with cerebral venous sinus thrombosis after anticoagulant therapy

**DOI:** 10.1097/MD.0000000000026360

**Published:** 2021-07-02

**Authors:** Shi-Hui Meng, Jing-Hua Li, Li-Jun Zuo, Li-Min Feng

**Affiliations:** a Department of Obstetrics and Gynecology, Peking University People's Hospital; b Department of Obstetrics and Gynecology; c Department of Neurology, Beijing Tiantan Hospital, Capital Medical University, Beijing, China.

**Keywords:** cerebral venous sinus thrombosis, efficacy, low molecular weight heparin, postpartum, pregnancy, safety

## Abstract

**Background:**

To describe the outcome of the patients with cerebral venous sinus thrombosis (CVST) during pregnancy and postpartum treated with anticoagulant therapy.

**Methods:**

This is a retrospective cohort study and patients with CVST were collected from October 2009 to March 2018. Patients were divided into pregnancy-related (occurred during pregnancy and postpartum) group and non-pregnancy-related. Recovery rate at 12 months after anticoagulant therapy, adverse events, characteristics of patients with poor outcomes were statistically analyzed.

**Results:**

Fifty-eight pregnancy-related CVST patients (17 pregnancy and 41 postpartum) as study group and 76 non-pregnancy-related CVST women as control group were enrolled. Study group was statistically different to control group in several baseline variables. More pregnancy-related patients had modified rankin scale (mRS) = 5 (15.5% vs 11.8%, *P* = 8.1×10^−3^) before anticoagulant therapy. At 12 months heparinization, difference in recovery rate was not statistically significant (80% vs 87.5%, *P* = .29) between 2 groups. No differences were found of adverse events between 2 groups. Patients with poor outcomes had less sigmoid sinus thrombosis (16.7% vs 61.5%, *P* = .14), more coma (41.2% vs 17.2%, *P* = 5.2×10^−7^), more mRS = 4 (33.3% vs 19.2%, *P* = 1.63 × 10^−4^), more mRS = 5 (66.7% vs 9.6%, *P* = 1.63 × 10^−4^) before treatment.

**Conclusion:**

Pregnancy-related CVST patients had severer condition before treatment, but can achieve comparable recovery rate at 12 months after anticoagulant therapy with non-pregnancy-related women. Pregnancy-related patients with poor prognosis had less sinus sigmoid occlusion, more coma, high mRS at admission.

## 1 Introduction

Cerebral Venous Sinus Thrombosis (CVST) is a relatively rare subtype of stroke which accounts for 4 to 6 per million people every year.^[1]^ Women are 3 times more likely to suffer from CVST due to hormonal changes, especially during postpartum and pregnancy.^[2]^ In a multicenter retrospective study of 465 women with CVST, 17% occurred during pregnancy and puerperium,^[3]^ sometimes causing poor maternal and fatal outcomes. Many survivors of CVST spent their lifetime with disability, bringing tremendous cost to their families.

Anticoagulants are the standard treatment for cerebral venous thrombosis, low-molecular-weight heparin (LMWH) are recommended in the guidelines.^[4]^ However, there remain doubts of LMWH in pregnancy and postpartum population with CVST. On one hand, anticoagulant therapy has the doubt of promoting intracerebral hemorrhages by causing hemorrhagic transformation of venous infarcts, which is more often in pregnancy and postpartum, and extra cerebral hemorrhage.^[5]^ On the other hand, anticoagulants always carry risks of vaginal bleeding, which may have higher incidence during normal pregnancy.^[6–8]^

Few data are available regarding the outcomes of CVST in mothers during pregnancy and postpartum. Furthermore, there is a lack of evidence to mid-term recovery rates and adverse events of LMWH therapy in this population.

Therefore, we conducted a retrospective study to depict a more complete picture of the real-world clinical recovery rates and to describe the outcomes of pregnancy-related CVST patients after anticoagulants.

## 2 Study methods

### 2.1 Study design and patient selection

We conducted this retrospective cohort study in Beijing Tiantan Hospital in China between October 2009 and March 2018, one of the largest medical and clinical research centers for neurological diseases. The research was approved by the Medical Ethics Committee of Beijing Tiantan Hospital, Capital Medical University (No. KY 2018-092-02) with informed consents obtained from patients or their consignors.

We enrolled patients based on the following eligibility criteria: female patients of childbearing age, diagnosed of CVST, the diagnosis was based on clinical manifestations, the diagnosis was made based on clinical manifestations and imaging. Imaging included contrasted computed tomography or contrasted magnetic resonance imaging, or magnetic resonance venography, computed tomography venography, or digital subtraction angiography, patients treated with low molecular weight heparin and warfarin. Patients were excluded if they received surgical interventions, they took combined oral contraception before onset, they had central nervous system infections, malignancy, traumatic brain injuries, and their data were incomplete. We excluded women taking combined oral contraception, one of the gender-specific risk factor for CVST,^[9]^ to get rid of the hormone fluctuation effects. Concomitant diseases that would influence modified rankin scale were excluded such as nervous system infections, malignancy, traumatic brain injuries. Patients suffered from CVST during pregnancy or within 6 months after delivery were divided into pregnancy-related group (study group), the rest were non-pregnancy-related group, served as control group. Study group was then divided into 2 subgroups based on dichotomized mRS (modified rankin scale) at 12 months: patients with severe poor outcome (mRS equals 5–6), patients without severe poor outcome (mRS≤4).

### 2.2 Study procedures and data collections

The management of patients was based on standard of care in affiliated hospitals of Capital Medical University. Patients were treated primarily with subcutaneous, low molecular weight heparin (Enoxaparin Sodium, Nadroparin Calcium, or Dalteparin Sodium) every 12 hours to maintain activated partial thromboplastin time between 1.5 and 2.5 times of control value. Enoxaparin sodium was used 100 Axa international unit/kg, nadroparin calcium was used based on patients’ weight:  < 50 kg 0.4 mL, 51 to 59 kg 0.5 mL, 60 to 69 kg 0.6 mL, dalteparin sodium was used 100 unit/kg. The last 3 days of heparization was combined with oral warfarin, then single warfarin was continually used to keep the international normalized ratio at 2 to 3 for at least 6 months. Patients were routinely asked to come to outpatient department for review 6 and 12 months after patients’ initial heparization, when doctors conducted mRS scoring and guided the use of oral warfarin, those who did not come were scored by phone interview made by neurologists.

Using an electronic medical record system and imaging system, the following data were collected for analysis: baseline information including age, gravidity, symptoms, risk factors, abnormal lab results, location of thrombosis, hemorrhagic lesion, venous infarct, intracranial pressure (ICP), mRS score before anticoagulant therapy and obstetric outcomes. Coma was defined as Glasgow coma scale less than 9. Severe ICP elevation was ICP≥330 mmH_2_O.

### 2.3 Outcome measurements

The primary outcome was the recovery rate after anticoagulant therapy at 12 months after heparization. Recovery rates were the percentage of patients had mRS 0 or 1 at follow-up. We adopted mRS because it can better evaluate patients’ ability to participate in the society after treatment. The secondary outcomes were: adverse events of anticoagulant therapy, differences between pregnancy-related patients with and without poor outcomes. Adverse events were defined according to the Common Terminology Criteria for Adverse Events (version 5.0): intracranial hemorrhage, including newly onset intracranial hemorrhage and expansion of pre-existing intracranial hemorrhage, vaginal bleeding, a fall in hemoglobin of or more than 2 g/dL, alanine aminotransferase (ALT) elevation, defined as ALT elevated at least 3 times than normal baseline or 2 times higher than abnormal baseline on first admission, gastrointestinal reaction, infection, defined as white blood cell higher than10 × 10^9^/L, or with detectable infectious foci.

### 2.4 Statistical analysis

Based on previous real-world study, the incidence of recovery was observed 94%^[10]^ in pregnancy-related group and 73.9%^[11]^ in non-pregnancy-related women. Fifty-one patients in each group could achieve more than 80% power to a significance level of 0.05 2-tailed. Considering 10% drop-out, 57 cases was a reasonable least sample size.

Baseline characteristics and laboratory results were summarized for 2 groups by means of descriptive statistics. Because data were not normally distributed, interquartile range was used for quantitative variable, and Mann–Whitney *U* test was used to compare group differences. For categorical variables, the *χ*^2^ test and the Kruskal–Wallis rank sum test were used for group comparisons. A propensity-matched analysis was also applied. Pregnancy-related group was matched with non-pregnancy-related group 1:1 based on the variable of age. Subjects who were not matched in non-pregnancy group were also deleted. Significance level was set at *P* < .05. We indicate in the tables if the probability values were corrected by false discovery rate correction for multiple testing. All data were analyzed by SPSS 23.0 (SPSS, IBM, NYU).

## 3 Results

### 3.1 Study population

**Figure 1:**
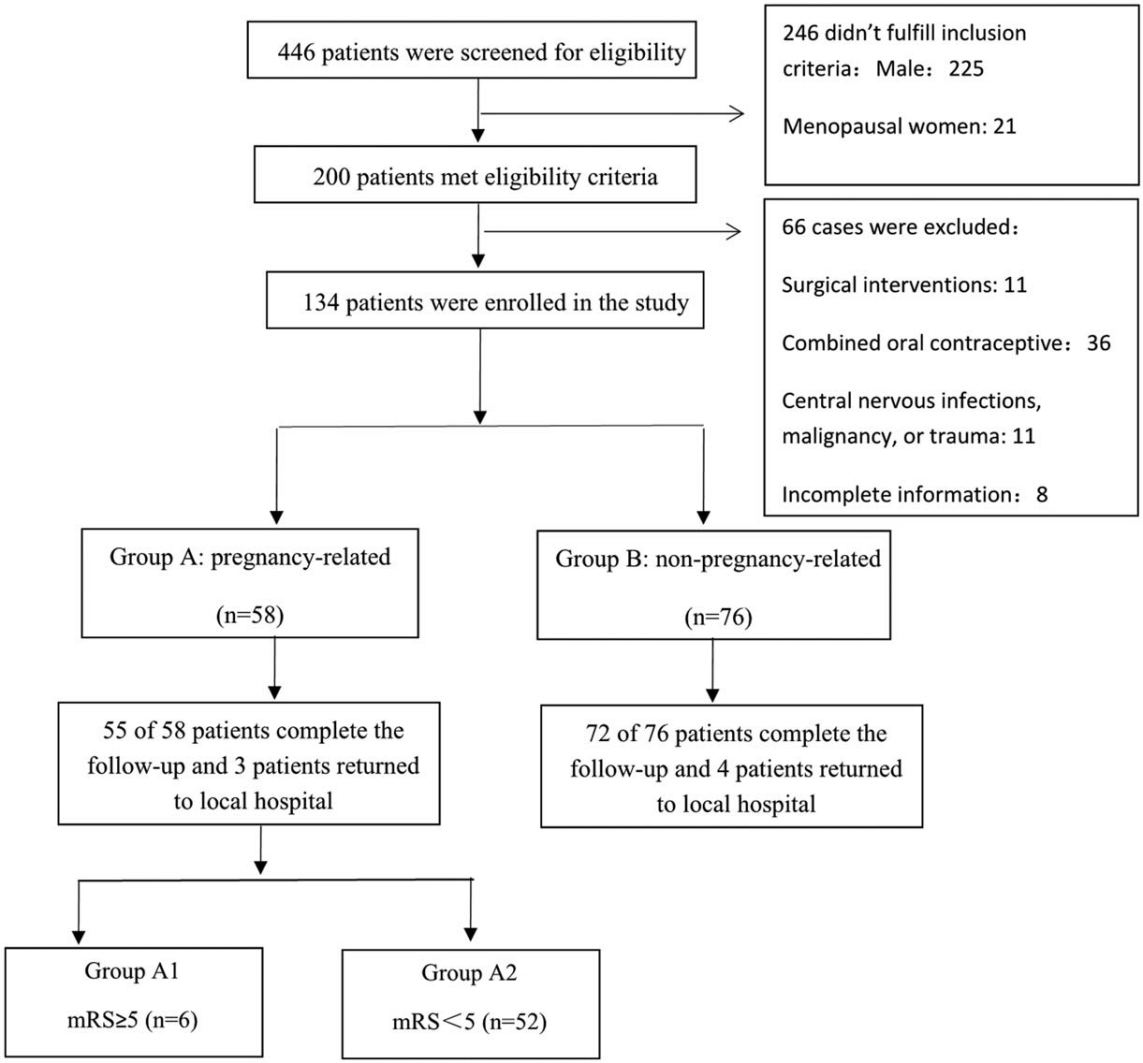
Disposition of patients. CVST = cerebral venous sinus thrombosis, LMWH = low-molecular-weight heparin, mRS = modified rankin scale.

We screened 446 patients, 200 of them met eligibility criteria. Sixty-six patients were excluded for intra-arterial thrombolysis (5 patients), intra-arterial thrombolysis combined with thrombectomy (4 patients), decompressive craniectomy (2 patients), received taking combined oral contraceptive before CVST (36 patients), suffering from central nervous infections, malignancy, or trauma (11 patients), incomplete information (8 patients). Finally, 134 patients were enrolled, including 58 pregnancy-related patients (17 during pregnancy and 41 during postpartum) and 76 non-pregnancy-related women (Fig. 1). Three pregnancy-related-patients and 4 non-pregnancy-related patients returned to the local hospital for rehabilitation therapy after discharge, they were not included in the 6  and 12 months outcomes analysis.

In baseline characteristics analysis, pregnancy-related patients were younger than non-pregnancy-related patients. Pregnancy-related patients also had a higher frequency of several initial symptoms and intracranial complications compared with non-pregnancy-related patients (Table 1).

**Table 1:** Baseline characteristics of pregnancy-related CVST patients and controls.

	Group A Pregnancy-related (n = 58)	Group B No-pregnancy-related (n = 76)	*P* value
Age (yr, IQR)	28 (24.8–32)	38.5 (29–45)	Z = 5.0, *P* < .01
Previous delivery, n (%)	29 (48.3)	38 (51.3)	χ^2^ = 0.1, *P* = .73
Initial symptoms, n (%)			
Headache	51 (87.9)	54 (71.1)	χ^2^ = 5.5, ^∗^*P* = .03
Nausea/vomiting	36 (62.1)	33 (43.4)	χ^2^ = 4.6, ^∗^*P* = .04
Seizures	33 (56.9)	14 (18.4)	χ^2^ = 21.4, ^∗^*P* < .01
Focal neurological deficit	35 (60.3)	21 (27.6)	χ^2^ = 14.5, ^∗^*P* < .01
Fever	17 (29.3)	4 (5.3)	χ^2^ = 14.4, ^∗^*P* < .01
Coma (GCS<9)	10 (17.2)	8 (10.5)	χ^2^ = 1.3, ^∗^*P* = .26
Etiology, n (%)			
Prothrombotic state	19 (32.8)	9 (11.8)	χ^2^ = 8.8, ^∗^*P* < .01
Infection	28 (48.3)	40 (52.6)	χ^2^ = 0.3, ^∗^*P* = .62
Autoimmune diseases	0 (0)	3 (2.2)	χ^2^ = 0.9, ^∗^*P* = .52
Abnormal lab results, n (%)			
Hyperhomocystinemia	12 (20.7)	8 (10.5)	χ^2^ = 2.7, *P* = .10
Anemia	20 (34.5)	30 (39.5)	χ^2^ = 0.4, *P* = .55
Hyperlipoidemia	28 (48.3)	23 (30.3)	χ^2^ = 4.5, *P* = .03
Location, n (%)			
Transverse sinus	45 (77.6)	62 (81.6)	χ^2^ = 0.3, ^∗^*P* = .43
Superior sagittal sinus	37 (63.8)	30 (39.5)	χ^2^ = 7.8, ^∗^*P* < .01
Sigmoid sinus	34 (58.6)	58 (76.3)	χ^2^ = 7.8, ^∗^*P* < .01
Straight sinus	9 (15.5)	10 (13.2)	χ^2^ = 0.2, ^∗^*P* = .70
Severe ICP elevation (ICP≥330 cm H_2_O), n (%)	12/35 (34.3)	27/61 (44.3)	χ^2^ = 0.9, *P* = .34
Intracranial complication, n (%)			
Intracranial hemorrhage	32 (55.2)	17 (22.4)	χ^2^ = 15.3, ^∗^*P* < .01
Cerebral infarction	27 (46.6)	18 (23.7)	χ^2^ = 7.7, ^∗^*P* < .01

CVST = cerebral sinus and venous thrombosis, GCS = glasgow coma scale, ICP = intracranial pressure, mRS = modified rankin scale.

∗ *P* was corrected by false discovery rate correction.

### 3.2 The recovery rate and adverse events of anticoagulant treatment in patients with CVST

**Figure 2:**
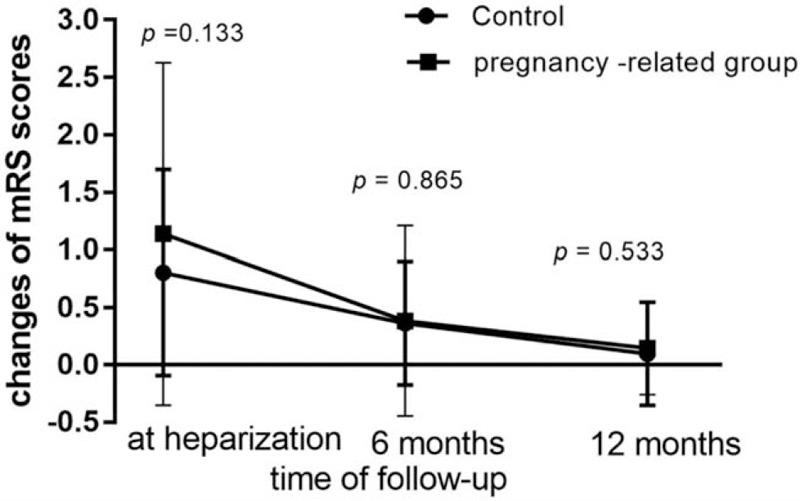
The change of mRS scores. mRS = modified rankin scale.

Before anticoagulant therapy, more pregnancy-related patients had mRS = 5 (15.5%), less patients had mRS = 2 to 4 (56.9%), as compared with control group. Therefore, when achieving heparinization, less recovery rate was found in study group (58.6%) than in control group (76.3%) (Table 2). However, recovery rate in study group gradually increased and at 12 months with no statistical significances between 2 groups in per-protocol analysis (80% vs 87.5%, *P* = .29). This trend is consistently shown in Figure 2, the decrease of mRS was larger in study group than control group at heparization, 6 and 12 months, but none of them had statistical significance. Similarly, disability rate (mRS 2–5) of study group was higher at heparization, but there were no statistical significances at 6 months and at 12 months between 2 groups. There were 5 deaths (10.4%) in study group and 2 (3%) in controls at the time of heparization. There were no statistical significances between 2 groups in the adverse events (Table 2), including intracranial hemorrhage and vaginal bleeding.

**Table 2:** The outcomes and adverse events of anticoagulation therapy in patients with cerebral sinus and venous thrombosis.

n (%)	Group A Pregnancy-related (n = 58)	Group B Non-pregnancy-related (n = 76)	*P* value
mRS score before anticoagulant therapy, n (%)	n = 58	n = 76	
0–1, n (%)	16 (27.6)	41 (53.9)	Z = 9.6, *P* < .01
2–4, n (%)	33 (56.9)	26 (34.2)	
5, n (%)	9 (15.5)	9 (11.8)	
Outcomes of patients after heparinization	n = 58	n = 76	
Complete recovery (mRS^∗^ 0–1)	34 (58.6)	58 (76.3)	Z = 2.3, *P* = .02
Disability (mRS 2–5)	19 (32.8)	16 (21.1)	
Death (mRS 6)	5 (8.6)	2 (2.6)	
Outcomes of patients at 6 mo heparinization	n = 55	n = 72	
Complete recovery (mRS 0–1)	40 (72.9)	60 (81.8)	Z = 1.6, *P* = .12
Disability (mRS 2–5)	10 (16.7)	10 (15.2)	
Death (mRS 6)	5 (10.4)	2 (3)	
Outcomes of patients at 12 mo heparinization	n = 55	n = 72	
Complete recovery (mRS 0–1)	44 (80.0)	63 (87.5)	Z = 2.5, *P* = .29
Disability (mRS 2–5)	6 (10.9)	7 (9.7)	
Death (mRS 6)	5 (9.1)	2 (2.8)	
Adverse events during treatment	n = 58	n = 76	
Intracranial hemorrhage	2 (3.4)	1 (1.3)	χ^2^ = 0.1, *P* = .81
Vaginal bleeding	3 (5.2)	0 (0)	χ^2^ = 2.0, *P* = .16
HGB decrease^†^	4 (6.9)	2 (2.6)	χ^2^ = 0.6, *P* = .45
ALT elevation^‡^	10 (17.2)	16 (21.1)	χ^2^ = 0.3, *P* = .58
Gastrointestinal reaction	2 (3.4)	4 (5.3)	χ^2^ = 0.01, *P* = .94
Infection	0 (0)	4 (5.3)	χ^2^ = 1.6, *P* = .21

∗ mRS = modified rankin scale.

† Caused a fall in hemoglobin of or more than 2 g/dL.

‡ ALT elevated at least 3 times than normal baseline or 2 times higher than abnormal baseline.

To further verify the outcomes of pregnancy-related CVST patients after anticoagulant treatment, we matched pregnancy-related group with non-pregnancy-related group based on the variable of age with PSM (Supplemental Table 1, which illustrates baseline characteristics after PSM). After matching, no differences were shown in mRS before treatment and at heparization. No differences were found of mRS at 6 months and at 12 months between 2 groups. However, compared with control group, more vaginal bleeding and less infections were found in pregnancy-related group. There were no severe adverse events (Supplemental Table 2, which illustrates outcomes and adverse events after PSM).

### 3.3 Comparison of pregnancy-related patients with and without severe poor outcome

**Figure 3:**
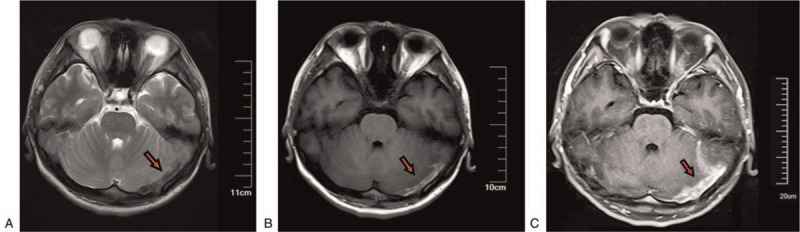
Images from pregnant women with transverse sinus thrombosis. A, T2-weighted image (T2WI) of MRI from a pregnant patient shows a clot in the left transverse sinus (red arrow). B, T1-weighted image (T1WI) of MRI from the same patient shows a clot in the left transverse sinus (red arrow). C, Contrast phase of MRI from the same patient shows a clot in the left transverse sinus (red arrow). MRI = magnetic resonance imaging.

Images of pregnant women with cerebral sinus thrombosis were shown in Figure 3. To evaluated differences between pregnancy-related patients with and without poor outcomes, we compared several variables (Table 3). There were 6 patients in poor outcome group, including 5 deaths and 1 persistent vegetative state. All patients with severe poor outcome had mRS 4 or 5 before treatment: 66.7% patients had mRS = 5, the other 33.3% had mRS = 4. In patients without severe poor outcome, only 9.6% patients had mRS = 5, 19.2% patients had mRS = 4, and the rest 71.2% had mRS = 0 to 3 before treatment. Patients with poor outcome also had more coma (Glasgow coma scale  < 9) and less sinus sigmoid occlusion.

**Table 3:** Comparison of pregnancy-related patients with and without poor outcomes.

	Patients with severe poor outcome (n = 6)	Patients without severe poor outcome (n = 52)	*P* value
Age (yr, IQR)	28.5 (23.3–37.4)	28 (25–31.75)	Z = 0.4, *P* = .71
Previous delivery, n (%)	3 (50)	23 (40.4)	χ^2^ = 0.01, *P* = .99
Symptoms, n (%)			
Headache	6 (100)	45 (86.5)	χ^2^ = 0.9, ^∗^*P* = .41
Nausea/vomiting	5 (83.3)	31 (59.6)	χ^2^ = 1.3, ^∗^*P* = .39
Seizures	2 (33.3)	31 (59.6)	χ^2^ = 1.5, ^∗^*P* = .33
Focal neurological deficit	2 (33.3)	33 (63.5)	χ^2^ = 2.0, ^∗^*P* = .45
Fever	2 (33.3)	15 (28.8)	χ^2^ = 0.1, ^∗^*P* = .82
Coma (GCS < 9)	6 (100)	4 (7.7)	χ^2^ = 32.1, ^∗^*P* < .01
mRS Score at admission, n (%)			
0–3	0	37 (71.2)	Z = 3.8, *P* < .01
4	2 (33.3)	10 (19.2)	
5	4 (66.7)	5 (9.6)	
Etiology, n (%)			
Infection	3 (50.0)	25 (48.1)	χ^2^ = 0.008, *P* = .93
Location, n (%)			
Transverse sinus	4 (66.7)	37 (71.2)	χ^2^ = 0.1, ^∗^*P* = .83
Superior sagittal sinus	5 (83.3)	32 (61.5)	χ^2^ = 1.1, ^∗^*P* = .58
Sigmoid sinus	1 (16.7)	32 (61.5)	χ^2^ = 4.4, ^∗^*P* = .14
Straight sinus	1 (16.7)	8 (15.4)	χ^2^ = .007, ^∗^*P* = .94
Intracranial Complications, n (%)			
Intracranial hemorrhage	5 (83.3)	27 (51.9)	χ^2^ = 2.2, ^∗^*P* = .14
Cerebral infarction	5 (83.3)	22 (83.3)	χ^2^ = 3.6, ^∗^*P* = .12

GCS = glasgow coma scale, IQR = interquartile range, mRS = modified rankin scale.

∗ *P* was corrected by false discovery rate correction.

Of 17 pregnant patients, 13 (76.5%) suffered in the first trimester, 2 (11.8) in the second trimester, and 2 (11.8%) in the third trimester. Median (interquartile range [IQR]) gestational week of onset was 9 (6.86–15.5) weeks. Precisely that 12 patients (70.6%) had abortion, 2 patients (11.8%) had live birth delivery, 3 patients (17.6%) died within 24 hours after admission and did not receive obstetrical interventions. Of 12 patients who underwent abortion, 10 patients had first-trimester abortion of manual vacuum aspiration, 2 patients had second-trimester abortion of intra-amniotic ethacridine lactate injection followed by misoprostol. Gestational week of first-trimester abortion was 8.43 (IQR, 6.75–9.68) weeks, no complication was found during or after vacuum aspiration surgery. In 2 patients received second-trimester abortion, at 23.14 and 18.14 weeks. 5% ethacridine lactate 100 mg was given and misoprostol 200 mg was given 2 days later. Three days after injection, 1 patient had a stillbirth of 20 cm in height and 40 mL of hemorrhage, she then received intrauterine curettage for retained fetal membranes. Another patient delivered a stillbirth of 23 cm with 50 mL of hemorrhage 4 days after injection. No complication was found in this patient. One patient's thrombosis vanished based on a neuroimage conformation at week 28. At week 38, this patient delivered a healthy boy with an Apgar score of 10 at 10 minutes and adequate weight by cesarean, required no incubators. Another patient was attacked during labor at week 41 and successfully delivered a healthy neonate. Mean days of onset were 12.5 (IQR, 6–22.25) days after delivery in postpartum patients. Of 41 postpartum patients, 22 (53.7%) patients had vaginal deliveries, 19 (46.3%) patients had cesarean. Reasons for cesarean were: scarred uterus (10 patients), severe pre-eclampsia (2 patients), oligohydramnios (2 patients, 1 of which delivered a neonate at 32 weeks weighed 1850 g), fetus distress (1 patient, neonate died after surgery), elective cesarean (4 patients).

## 4 Discussion

CVST is a rare disease with preference in pregnancy-related patients. This study showed differences about characteristics and outcomes between pregnancy-related CVST patients and non-pregnancy-related women who underwent low molecular weight heparin and consequent warfarin therapy.

Few studies compare the characteristic differences between pregnancy-related patients and non-pregnancy-related women in developing countries. In our study, pregnancy-related patients were younger with a higher frequency of prothrombotic state and hyperlipoidemia, headache, nausea or vomiting, seizures, focal neurological deficit, fever. Pregnant or postpartum patients with these symptoms visit outpatient department, doctors should be vigilant against CVST. Besides, most of the fever in pregnancy-related patients was higher than 38.5°C, which was not simply caused by puerperium breast engorgement, antibiotics should be used when necessary. Pregnancy-related patients had higher frequency of parenchymal lesions (intracranial hemorrhage: and cerebral infarction) that manifested as focal neurological deficit, seizures, coma, which may explain the higher mRS at admission in pregnancy-related group than control group. Also, some doctors are not aware of CVST in pregnant or postpartum patients. Diagnosis may be postponed and CVST may proceed during the process.

Pregnancy-related patients had severer condition at admission, but recovery rate was comparable to non-pregnancy-related patients at 12 months follow-up. Though at heparization, recovery rate was lower compared with control group, it gradually increased. At 12 months there was no statistical significance to control group. The results were still consistent after propensity score matching. In Figure 2 we can also see mRS in pregnancy-related group has changed more than control group in every follow-up point. Therefore, it can be inferred that pregnancy-related patients had stronger ability to recover and may have good prognosis in the long term. Previous retrospective studies reported that CVST in pregnant women has a relatively favorable prognosis. Stolz et al^[12]^ showed all 11 patients during partum or postpartum achieved mRS < 3 at 6 months follow-up (*P* < .05). However, this study enrolled men in the univariate analysis, who may have a poorer prognosis than women.^[4]^ Besides, this result is limited to the small sample size, and the univariate analysis could not control variables, many confounding factors such as male gender, hemorrhagic transformation of infarct, venous infarct, site of thrombosis may be involved. Another study^[13]^ with a larger sample of 67 pregnancy or puerperium patients showed 80.6% good outcome compared with 58.6% in patients of other causes. However, there was no standard measurement to “good outcome,” and the males also enrolled. Besides, there were lower ratio of stupor/com, seizures, and focal signs in the study group compared with the control group, meaning the control group had a severer situation at admission. In our study, pregnancy-related patients had more coma, focal neurological deficit, and seizure with obvious statistical significances.

Due to special physiological state during pregnancy and postpartum, there are concerns that anticoagulant therapy may cause more adverse events to this population. In this study, there were no differences of hemorrhagic events in both groups despite the high proportion of complicated intracranial hemorrhage in the pregnancy-related group. Besides, ALT elevation that may be related to LMWH treatment was common but with no differences between groups. This can suggest the safety of anticoagulant therapy in pregnancy-related patients. According to Chinese Stroke Association guidelines 2019,^[14]^ LMWH followed by warfarin is recommended throughout the pregnancy. However, obstetric management consequent to treatment remained unclear. A meta-analysis^[10]^ showed 17 pregnancies resulting in live births and 9 resulting in therapeutic abortion or unintended pregnancy loss. Safety of persistent pregnancy needs further investigation.

CVST in pregnancy-related patients can cause poor outcomes. Death rates of study group were similar with the previous study^[15]^ about puerperal CVT (manifesting within 1 month of delivery or abortion) (8/73, 11%) after heparin treatment. Both were higher than control group. In the subgroup analysis of pregnancy-related patients with poor outcomes, mRS at admission was higher in patients with poor outcomes (mRS = 4 or 5). This may suggest patient's situation before treatment may influence outcome, and patients with high mRS may need additional interventions such as neurosurgery. Furthermore, doctors should manage CVST on time, prevent it from progressing into severe situation, and therefore achieve favorable outcomes.

There are no randomized controlled trials or case-control studies assessing optimal duration of oral anticoagulation for the prevention of recurrent CVST. A retrospective study^[16]^ reported CVT recurrence in 23.6 events/1000 patient-years (95% CI, 17.8–28.7) after anticoagulant therapy withdrawal. In another study^[17]^ including 624 patients with CVT, 2.2% patients had a recurrent CVT and 4.3% had a VTE in other sites. 58.3% VTE and 64.3% CVT were on anticoagulation at the time of recurrence and 63% VTE occurred within the first year. In our study, the duration of oral anticoagulant therapy was 6–12 months. No recurrence of VTE or CVT was found, suggesting a low recurrence in female population.

There are several limitations in our study. First, the sample size was small. Based on the data of our trial, complete recovery rate was 58.6% in study group and 76.3% in control group. This could only achieve 70% power at the significance of.05 at 1-tailed and 57% power at 2-tailed. The least patients were 85 patients in each group at a significance level of 0.05 (1-tailed) and 80% power. However, CVST is a rare stroke with low incidence rate, data from large-scale trials and prospective multiple-centered studies are needed. Second, in more CVST patients complicated by intracranial hemorrhage are needed to evaluate anticoagulant therapy in those population. Third, 7 patients returned to local hospital for rehabilitation therapy, per-protocol analysis was performed at 6 and 12 months prognosis. At the same time, large-scale trials and prospective multiple-centered studies are needed.

## 5 Conclusions

Pregnancy-related CVST patients had severer condition at admission, but can achieve comparable recovery rate after anticoagulant therapy compared with non-pregnancy-related women. Anticoagulant therapy did not add safety concerns to pregnancy-related patients. Pregnancy-related patients with poor prognosis had less sinus sigmoid occlusion, more coma, high mRS at admission.

## Author contributions

Drs. Li and Feng proposed the concept and designed the study. Dr Meng contributed to the acquisition of data with the help of Dr Li. Dr. Meng performed the statistics and interpreted the data and wrote the manuscript with assistance from Drs. Feng and Zuo. All authors provided inputs for the manuscript. All authors read and approved the final manuscript.

**Conceptualization:** Limin Feng.

**Data curation:** Shihui Meng.

**Formal analysis:** Shihui Meng, Jinghua Li.

**Methodology:** Jinghua Li.

**Software:** Shihui Meng.

**Supervision:** Limin Feng.

**Validation:** Limin Feng.

**Writing – original draft:** Shihui Meng, Jinghua Li.

**Writing – review & editing:** Shihui Meng, Li-Jun Zuo, Limin Feng.

## Supplementary Material

Supplemental Digital Content

## Supplementary Material

Supplemental Digital Content
