# Supplementary material for: The outcomes of pregnant and postpartum patients with cerebral venous sinus thrombosis after anticoagulant therapy
Source: Medicine (Baltimore). 2021 Jul 2;100(26):e26360. doi: 10.1097/MD.0000000000026360 (PMC8257884; doi:10.1097/MD.0000000000026360)
Supplement: Supplemental Digital Content [file medi-100-e26360-s001.docx]

**Table 1: Propensity score matching for Baseline characteristics**

|  | **Matched Group A**  **Pregnancy-related**  **(n = 42)** | **Matched Group B**  **No-pregnancy-related**  **(n = 42)** | **P-value** |
| --- | --- | --- | --- |
| **Age (y, IQR)** | 29 (29-32.3) | 31 (31-36) | Z=0.3, P=0.73 |
| **Previous delivery, n (%)** | 20 (47.6) | 16 (38.1) | χ^2^=0.8, P=0.38 |
| **Initial symptoms, n (%)** | | | |
| Headache | 39 (92.9) | 31 (73.8) | χ^2^= 5.5, *P= 5.7x10^-3^ |
| Nausea/Vomiting | 27 (64.3) | 17 (40.5) | χ^2^= 4.8, *P=3.5 x10^-2^ |
| Seizures | 21 (50.0) | 7 (16.7) | χ^2^= 10.5, *P=5.8x10^-3^ |
| Focal neurological deficit | 22 (52.4) | 13 (31.0) | χ^2^= 4.0, *P=3.4x10^-2^ |
| Fever | 9 (21.4) | 2 (4.8) | χ^2^= 5.1, *P=4.8x10^-2^ |
| Coma (GCS<9) | 6 (14.3) | 4 (9.5) | χ^2^= 0.5, *P= 0.5 |
| **Etiology, n (%)** |  |  |  |
| Prothrombotic state | 15 (35.7) | 4 (9.5) | χ^2^=8.2, *P=1.2x10^-2^ |
| Infection | 21 (50.0) | 26 (61.9) | χ^2^=1.2, *P= 0.27 |
| Autoimmune diseases | 0 (0) | 4 (9.5) | χ^2^=4.2, *P=0.045 |
| **Abnormal lab results, n (%)** |  |  |  |
| Hyperhomocystinemia | 10 (23.8) | 4 (9.5) | χ^2^=3.1, *P=8.8x10^-2^ |
| Anemia | 17 (40.5) | 15 (35.7) | χ^2^=0.2, *P= 0.65 |
| Hyperlipoidemia | 22 (52.4) | 11 (26.2) | χ^2^=6.0, *P= 6.3 x10^-2^ |
| **Location, n (%)** |  |  |  |
| Transverse sinus | 39 (92.9) | 33 (78.6) | χ^2^=3.5, *P=0.24 |
| Superior sagittal sinus | 24 (57.1) | 18 (42.9) | χ^2^=1.7, *P=0.56 |
| Sigmoid sinus | 28 (66.7) | 30 (71.4) | χ^2^=0.2, *P=0.64 |
| Straight sinus | 7 (16.7) | 4 (9.5) | χ2=0.9, *P=0.30 |
| **Intracranial complication, n (%)** |  |  |  |
| Intracranial hemorrhage | 24 (57.1) | 8 (19.0) | χ^2^=12.9, *P=6.5x10^-4^ |
| Cerebral infarction | 19 (45.2) | 9 (21.4) | χ^2^=5.4, *P=2.1x10^-2^ |

**CVST: cerebral sinus and venous thrombosis; mRS: modified rankin scale; ICP: intracranial pressure.** ***p was corrected by false discovery rate correction.**
