# Supplementary material for: The outcomes of pregnant and postpartum patients with cerebral venous sinus thrombosis after anticoagulant therapy
Source: Medicine (Baltimore). 2021 Jul 2;100(26):e26360. doi: 10.1097/MD.0000000000026360 (PMC8257884; doi:10.1097/MD.0000000000026360)
Supplement: Supplemental Digital Content [file medi-100-e26360-s002.docx]

**table 2:** **Propensity score matching for outcomes and adverse events**

| **n (%)** | **Matched Group A**  **Pregnancy-related**  **(n =42)** | **Matched Group B**  **Non-pregnancy-related**  **(n=42)** | **P-value** |
| --- | --- | --- | --- |
| **mRS score before anticoagulant therapy, n (%)** | **n=42** | **n=42** |  |
| 0~1, n (%) | 15 (35.7) | 22 (52.4) | Z=759, P=0.22 |
| 2~4, n (%) | 24 (57.1) | 16 (38.1) |  |
| 5, n (%) | 3 (7.1) | 4 (9.5) |  |
| **Outcomes of patients after heparinization** | **n=42** | **n=42** |  |
| Complete Recovery (mRS^a^ 0-1) | 26 (61.9) | 32 (76.2) | Z=752, P=0.15 |
| Disability (mRS 2-5) | 12 (28.6) | 8 (19) |  |
| Death (mRS 6) | 4 (9.5) | 2 (4.8) |  |
| **Outcomes of patients at 6 months** **heparinization** | **n=39** | **n=39** |  |
| Complete Recovery (mRS 0-1) | 30 (76.9) | 33 (84.6) | Z=699, P=0.37 |
| Disability (mRS 2-5) | 5 (12.8) | 4 (10.3) |  |
| Death (mRS 6) | 4 (10.3) | 2 (5.1) |  |
| **Outcomes of patients at 12 months** **heparinization** | **n=39** | **n=39** |  |
| Complete Recovery (mRS 0-1) | 31 (79.5) | 34 (87.2) | Z=700, P=0.35 |
| Disability (mRS 2-5) | 4 (10.3) | 3 (7.7) |  |
| Death (mRS 6) | 4 (10.3) | 2 (5.1) |  |
| **Adverse events during treatment** | **n =42** | **n=42** |  |
| Intracranial hemorrhage | 1 (2.4) | 1 (2.4) | χ2=0, *P=1 |
| Vaginal bleeding | 3 (7.1) | 0 (0) | χ2=3.1, P=7.8x10^-3^ |
| GB decrease^b^ | 2 (4.8) | 0 (0) | χ2=2.0, P=0.15 |
| ALT elevation^c^ | 9 (21.4) | 8 (19.0) | χ^2^=0.7, P=0.79 |
| Gastrointestinal reaction | 2 (4.8) | 3 (7.1) | χ^2^=0.2, P=0.65 |
| Infection | 0 (0) | 4 (9.5) | χ^2^=4.2, P=0.04 |

**^a^mRS: modified rankin scale; ^b^Caused a fall in hemoglobin of or more than 2 g/dL; ^c^ALT elevated at least 3 times than normal baseline or two times higher than abnormal baseline.**
